# The use of factor analysis and abductive inference to explore students’ and practitioners’ perspectives of feedback: divergent or congruent understanding?

**DOI:** 10.1186/s12909-020-02378-w

**Published:** 2020-11-25

**Authors:** Christine Ossenberg, Amanda Henderson, Marion Mitchell

**Affiliations:** 1 Griffith University, School of Nursing and Midwifery, Kessels Road, Nathan, Queensland 4111 Australia; 2 Central Queensland University, Ann Street, Brisbane, Queensland 4000 Australia; 3 Menzies Health Institute Queensland, Griffith University, Brisbane, 4111 Australia

**Keywords:** Feedback; workplace-based, Student, Practitioner, Learner, Learning partner

## Abstract

**Background:**

The importance of feedback in workplace-based settings cannot be underestimated. Approaches that evaluate feedback reflect either the sender’s or receiver’s viewpoint in isolation of each other. This study investigated prevailing student and practitioner views of feedback resulting from development and testing of a survey about feedback.

**Method:**

This study used a cross-sectional design, incorporating use of expert consultation and factor analysis of surveys. Fifty-two items based on identified attributes for effective feedback from current research were developed and reviewed through expert consultation. Surveys developed from the items were completed by students (*n* = 209) and practitioners (*n* = 145). The juxtaposition of items based on students’ and practitioners’ responses to the surveys were examined through use of exploratory factor analysis.

**Results:**

Separate student and practitioner surveys resulted. Each survey contained 23 items that clustered into factors. The item statements were different across practitioner and student groups Only nine items were shared across factors identified for both groups. The resulting factors represented different notions of feedback—namely, practitioners had a process-oriented focus in comparison with students’ outcome focus.

**Conclusion:**

While students and practitioners view feedback differently this does not necessarily mean they are incongruous.

**Supplementary Information:**

The online version contains supplementary material available at 10.1186/s12909-020-02378-w.

## Background

Feedback is a core component of the educational process [1] in both academic and workplace-based settings. The importance of feedback in workplace-based settings cannot be underestimated. Workplace-based settings provide learners the opportunity to acquire discipline specific skills and knowledge as well as develop linguistic and discourse patterns particular to their profession [2]. As such effective feedback on workplace-based performance is a key element in helping the learner to develop capacity, to evaluate their performance, and change behaviours [3, 4]. However, determining the effectiveness of feedback can be challenging as the quality of feedback is variable [5] with learners commonly reporting they still receive little feedback [1] – despite the abundance of literature focused on feedback.

Current conceptualisations describe feedback as dialogic [6, 7]. That is, feedback “…involves relationships in which participants think and reason together” [7] (p.286). An assumption also exists that learners (e.g. students) and learning partners (i.e. someone who supports a learner in the feedback process, for example practitioners) share a common understanding of the term ‘feedback’ [3]. If learners and learning partners do not share the same understanding of feedback, then the commonplace approach to examine one-sided viewpoints of effective feedback must be questioned. Investigating feedback drawing on empirical findings could assist to substantiate conceptual understanding of feedback proffered in the extant literature.

Literature exploring approaches that evaluate the prevailing discourse of feedback is in its infancy. Halman et al. [5] developed and described validity evidence for the Direct Observation of Clinical Skills Feedback Scale (DOCS-FBS). This instrument is specifically intended to rate the quality of verbal feedback provided by assessors in the clinical environment and was tested through participants using the scale to rate videotaped feedback interactions. Bing-You et al. [1] present validity evidence for two Feedback in Medical Education (FEEDME) instruments for use in the clinical setting. The FEEDME-Culture instrument is completed by the learner and developed to assess medical students’ and residents’ perceptions of the feedback they receive. The FEEDME-Provider instrument is a companion instrument also completed by the learner. This instrument aims to ascertain the medical students’ and residents’ perceptions of how the faculty member provided feedback.

Both the DOCS-FBS and the FEEDME instruments focus on the feedback received in the clinical setting: the DOCS-FBS based on a one-off feedback interaction; and the FEEDME based on feedback encounters during a clinical rotation. Neither, however, provide insight into the learning partner’s perception of the feedback quality in tandem with the learner’s perception. Therefore, there is value in development of instruments that explore effective feedback from the views of both the learner and learning partner and exploring the meaning of any eventuating structural analysis.

### Aim

This study explored prevailing student and practitioner perspectives of feedback through instrument validation and structural analysis of the Quality Feedback Inventory (QFI) and ‘sense-making’ of the resultant factors.

### Ethical considerations

Approval to conduct this study was obtained from the Human Research Ethics Committees of the university (Reference number: 2018/341) and health care service (HREC/18/QPAH/93) where the study was conducted. Participant information outlined the purpose and anticipated benefits of this study. Participation was voluntary; with the return of completed or partially completed surveys taken as an indication of respondents’ consent to participate. Surveys were anonymous and therefore non-identifiable to the research team.

## Method

**Fig. 1:**
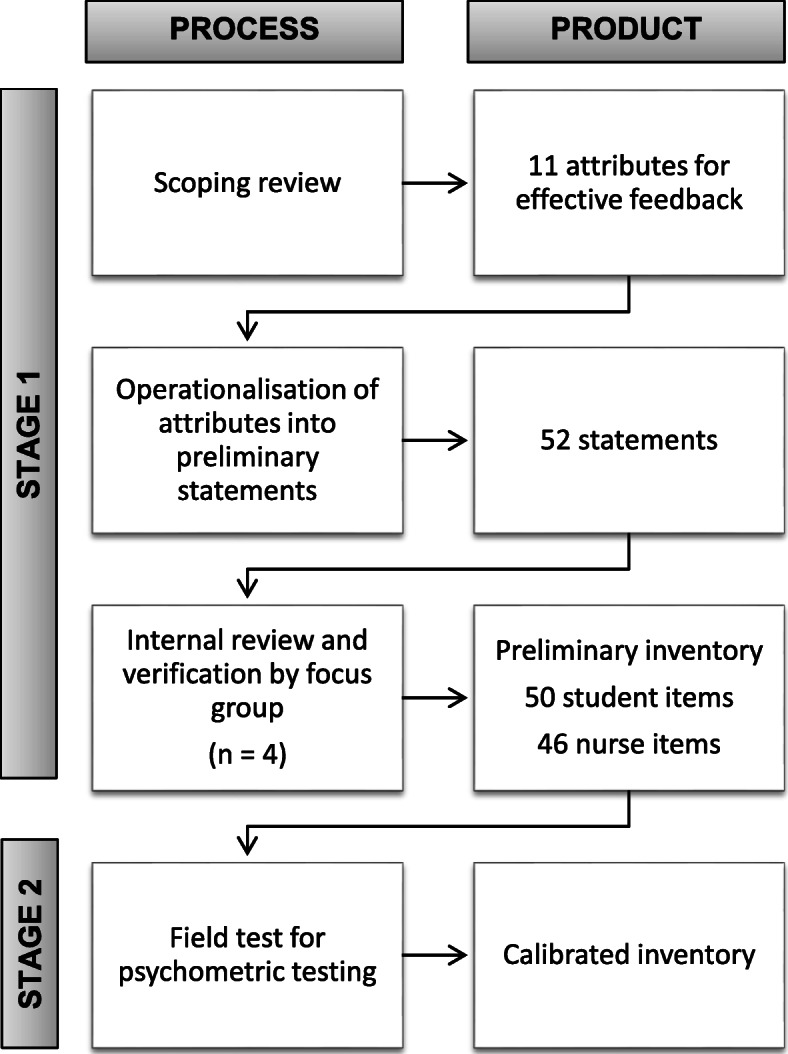
Flow chart of inventory development process and product

This study used a cross-sectional design, incorporating a focus group technique and factor analysis of surveys, to compare and contrast student and practitioner views of feedback. It draws on empirical data exploring the value of feedback. The study involved two stages: stage one, the generation of items for a list that explores views of feedback; and stage two, data collection and explanatory factor analysis of the list of items (see Fig. 1).

### Stage one – item generation

Stage one was conducted throughout June 2018. The use of research findings as a source of items has been identified as an effective approach in item generation [8]. Therefore, a range of simple statements based on recent scoping review findings that identified 11 key attributes of feedback [9] (see Tables 1 and 2) and existing feedback instruments (e.g. FEEDME-Culture, DOCS-FBS) were crafted by the research team. Constructed statements were discussed and verified by the research team at a face to face meeting. Inclusion of statements was achieved through majority consensus. Generation of a large item pool relevant to the concept of interest is preferential and strengthens the internal-consistency reliability (and therefore validity) of the emerging scale [10]. An initial 52 items were developed and organised into two lists of items that provided individualised item language for students and practitioners. For example, the item regarding evaluating practice became ‘I was encouraged to evaluate my own practice’ for the student list of items and ‘I encouraged the student to evaluate their practice’ for the practitioner list of items.

**Table 1:** Initial 46 practitioner items and associated feedback attributes

Items – Practitioner inventory^**a**^		Feedback Attribute^**b**^
**1.1**	**I encouraged the student to evaluate their practice**	Process
1.2	I encouraged the student to reflect on their practice^c^	
1.3	I encouraged the student to share their reflection as part of the feedback process^c^	
**2.1**	**The learning goals were agreed in advance with the student**	Criteria-based
**2.2**	**Feedback was related to workplace or university standards**	
*2.4*	*Feedback was relevant to what was expected of the student*	
*3.1*	*Feedback was based on direct observation of the student’s practice*	Multiple forms and sources of evidence
**3.2**	**Feedback informed by multiple sources**	
4.1	The student asked for feedback^c^	Desired
4.2	The student appreciated the feedback^c^	
*4.3*	*The student welcomed the feedback*	
**5.1**	**There was enough time for feedback**	Timely
**5.3**	**Feedback was timely**	
**5.4**	**Feedback occurred at an agreed time**	
*6.1*	*Feedback was specific to the learning needs of the student*	Responsive to the learner
**6.2**	**Feedback was relevant to the student’s situation**	
**6.3**	**I encouraged the student to be involved in feedback conversations**	
*6.5*	*The student had the opportunity to clarify feedback*	
**6.6**	**I encouraged the student to ask questions to help them understand the feedback**	
*6.7*	*The student understood the feedback*	
**7.1**	**The amount of feedback was manageable**	Frequent
**7.2**	**Feedback was regular**	
*7.3*	*Feedback was planned*	
**7.4**	**Feedback was expected**	
8.1	Learning goals were reviewed based on feedback^c^	Future-focused
**8.2**	**Learning goals were modified based on feedback**	
**8.4**	**Feedback helped the student to know how to improve their practice**	
**9.1**	**I felt the student was comfortable sharing their viewpoints**	Reciprocal
*9.2*	*I felt I listened to the student*	
*9.3*	*Feedback was communicated in a way the student could understood*	
*9.4*	*I adapted my communication style to meet the student’s learning needs*	
**10.1**	**The feedback I shared was respectful**	Skilful interaction
**10.2**	**The feedback I shared was clear**	
**10.3**	**The feedback I shared was non-judgemental**	
**10.4**	**The feedback I shared was non-threatening**	
*10.5*	*The feedback I shared focused on student’s practice*	
**10.6**	**I considered the student’s emotional needs**	
10.7	The feedback I shared addressed specific areas of the student’s practice^c^	
*10.8*	*The feedback I shared was specific not generalised*	
*11.2*	*The feedback I shared was offered in different ways*	Multidimensional
11.4	The feedback I shared focused on the student’s knowledge^c^	
*11.5*	*The feedback I shared focused on the student’s performance*^c^	
*11.6*	*I considered the student’s decision making process*	
**11.7**	**Feedback encouraged the student to share their feelings about different experiences**	
*11.8*	*Feedback encouraged the student to think about how their individual beliefs/values influenced their practice*	
**11.9**	**Feedback encouraged the student to think about what motivates them to learn**	
12.1	I believe feedback is important	Global items
12.2	I clearly identified to the student I was communicating feedback regarding their performance	
12.3	Overall, I believe the feedback I shared reinforced the student’s practice	
12.4	Overall, I believe the feedback I shared helped change the student’s practice	
12.5	Overall, I believe the feedback I shared made the student think differently about their practice	

^a^ Items 2.3, 5.2, 8.3, 11.1, 11.3 removed prior to analysis as non-comparable item; item 6.4 removed – duplicate of item 10.2

^b^ Ossenberg, C., Henderson, A., & Mitchell, M. (2019). *What attributes guide best practice for effective feedback? A scoping review*. Advances in Health Sciences Education, 24 (2):383–401

^c^ Initial item removal based on correlation ≤ .2

Items retained in the final 23-item student inventory are in **bold**

Items eliminated from the final 23-item student inventory are in *italics*

**Table 2:** Initial 50 student items and associated feedback attributes

Items – Student inventory^**a**^		Feedback Attribute^**b**^
*1.1*	*I was encouraged to evaluate my own practice*	Process
**1.2**	**I was encouraged to reflect on my evaluation**	
*1.3*	*I had an opportunity to share my reflection as part of the feedback process*	
*2.1*	*The learning goals for my practice were agreed in advance with my assessor/nurse*	Criteria-based
*2.2*	*Feedback was based on the agreed learning goals*	
**2.3**	**Feedback related to specific standards**	
*2.4*	*Feedback was relevant to what was expected of me*	
**3.1**	**Feedback was informed by observation of my practice**	Multiple forms and sources of evidence
*3.2*	*Feedback was informed by multiple sources*	
4.1	I asked for feedback^3^	Desired
*4.2*	*I appreciated the feedback*	
*4.3*	*Feedback was welcomed*	
**5.1**	**There was enough time for feedback**	Timely
*5.2*	*Feedback was not rushed*	
*5.3*	*Feedback was timely*	
**5.4**	**Feedback occurred at an agreed time**	
*6.1*	*Feedback was specific to my learning needs*	Responsive to the learner
**6.2**	**Feedback was relevant to my situation**	
**6.3**	**I was encouraged to be involved in feedback conversations**	
**6.5**	**I had the opportunity to clarify feedback**	
*6.6*	*I was encouraged to ask questions to help me understand the feedback*	
**6.7**	**I understood what the feedback meant**	
*7.1*	*The amount of feedback was manageable*	Frequent
*7.2*	*Feedback was regular*	
**7.3**	**Feedback was planned**	
*7.4*	*Feedback was expected*	
**8.1**	**Learning goals were reviewed based on feedback**	Future-focused
8.2	Learning goals were modified based on feedback^c^	
**8.3**	**Feedback motivated me to change**	
**8.4**	**Feedback helped me to know how to improve my practice**	
**9.1**	**I felt comfortable sharing my opinion/viewpoint**	Reciprocal
*9.2*	*I felt I was listened to*	
**9.3**	**Feedback was communicated in a way I understood**	
**10.1**	**Feedback was respectful**	Skilful interaction
**10.2**	**Feedback was clear**	
**10.3**	**Feedback was non-judgemental**	
*10.4*	*Feedback was non-threatening*	
**10.5**	**Feedback focused on my practice**	
**10.6**	**My emotional needs were considered**	
*10.7*	*Feedback was specific not general*	
*10.8*	*Feedback addressed specific area/s of my practice*	
**11.1**	**Feedback was offered in more than one way**	Multidimensional
*11.2*	*Feedback was offered in different ways*	
*11.3*	*Feedback was offered at multiple times*	
**11.4**	**Feedback focused on my knowledge**	
*11.5*	*Feedback focused on my performance*	
**11.6**	**My decision making process was considered**	
*11.7*	*Feedback encouraged me to share my feelings about different experiences*	
*11.8*	*Feedback encouraged me to think about how my individual beliefs/values influenced my practice*	
*11.9*	*Feedback encouraged me to think about what motivates me to learn*	
12.1	Feedback is important to me	Global items
12.2	The communication regarding my performance was labelled as ‘feedback’	
12.3	Overall, the feedback shared reinforced my practice	
12.4	Overall, the feedback helped me change my practice	
12.5	Overall, the feedback made me think differently about my practice	

^a^ Item 9.4 removed prior to analysis as non-comparable item; item 6.4 removed – duplicate of item 10.2

^b^ Ossenberg, C., Henderson, A., & Mitchell, M. (2019). *What attributes guide best practice for effective feedback? A scoping review*. Advances in Health Sciences Education, 24 [2]:383–401

^c^ Initial item removal based on correlation ≤ .2

Items retained in the final 23-item student inventory are in **bold**

Items eliminated from the final 23-item student inventory are in *italics*

Seeking expert opinion is a useful approach to determine whether ideas or constructs of interest make sense [8] and can be conducted after item development to provide evaluative judgement “regarding the content representativeness (relevance, accuracy, and completeness) of the selected items…” [11] (p.63). A group discussion of four experienced nurses, who support student learning in clinical learning environments and experienced in giving and receiving feedback, was conducted. An explanation of the underpinning feedback attribute for each item was provided to the group. Members of the expert consultation group reviewed each item to establish its clarity and relevance to the underlying attribute. This process facilitated a discussion on item interpretation through cognitive probes such as ‘What do you understand by item X?’, ‘What does item X mean to you?’ or ‘How else could you phrase this item?’. Additionally, the expert group participants checked the wording of each item to ensure it reflected common phrasing in the workplace. Changes to the item wording was made immediately and redundant items removed. All changes were verified by the group to ensure consensus was achieved. This resulted in a preliminary pool of 50 items in the student survey and 46 items in the practitioner.

### Stage two – collection and analysis of surveys

#### Procedure

Stage two of the study involved distribution of the two surveys and was conducted from July to November 2018 at two teaching healthcare facilities in South East Queensland, Australia. Participants were students and practitioners. The student group were third-year nursing students on a four-week clinical placement. The practitioner group were nurses positioned to assist student learning in the clinical learning environment and therefore expected to be involved in providing feedback. All participants were invited to complete the surveys towards the end of the four-week clinical placement period during which students and practitioners were involved with multiple feedback encounters. Students and practitioners scored items on a five-point Likert scale (1 = never, 2 = rarely, 3 = sometimes, 4 = often, and 5 = always).

#### Analysis

An independent research assistant entered all data into an electronic spreadsheet prior to analysis and therefore non-identifiable to the researchers. Data were transferred into the International Business Machines Corporation Statistical Package for Social Sciences (IBM-SPSS version 25 for Windows); screening for errors and other anomalous data was undertaken prior to analysis. Cases were removed from analysis if ≥50% of responses were incomplete. We examined skewness and kurtosis at item level to determine if assumptions of normality were met [12].

An initial principal component analysis (PCA) and subsequent exploratory factor analysis (EFA) was performed (using principal axis factoring and oblique rotation) to determine which items possibly correlated creating a ‘factor’ [12]. Factorability of the list of items was determined by examining the Kaiser-Meyer-Olkin (KMO) test of sample adequacy and Bartlett’s test of sphericity. We also used the eigenvalue greater than one criterion [13, 14] and scree test to determine the number of factors.

## Results

### Participants

Surveys were completed by 425 participants: nursing students (*n* = 239), and practitioners (*n* = 186). Females represented 85% of participants, males represented 12.5%, and non-binary 0.5%, with 2% of surveys not having gender recorded. The mean age of nursing students was 24 years (SD = 4.9) and of practitioners 36 years (SD = 11.8). Practitioners reported an average of 6.5 years (SD 6.8) experience supporting students in the workplace.

### Construct validity

Where possible, the surveys were designed with comparable items across student and practitioner groups. An initial PCA was undertaken to appraise factorability of the correlation matrix and to establish which components exist within the data [13]. Cases were excluded list-wise to ensure a valid case on every variable for every case [13–16]. PCA was run separately on the 46-item practitioner (*n* = 145) and 50-item student (*n* = 209) surveys using orthogonal (varimax) and oblique (direct oblimin) rotation. There was minimal difference between rotation strategies. Direct oblimin rotation was selected as items in the lists were focused on a common construct (i.e. feedback) as one would logically expect some degree of correlation between factors.

The Kaiser-Meyer-Olkin (KMO) measure of sampling adequacy was > .9 for items in both student and practitioner surveys. The KMO values for individual items (anti-image matrix) was > .77 – both above accepted minimum of .6 – also supporting factorability of the items in the surveys [13]. Bartlett’s test of sphericity was significant for the student (χ^2^(253) = 3714.45, *p* < .001) and practitioner items (χ^2^(253) = 1656.93, p < .001). Scatterplots confirmed linearity of data. Interrogation of individual matrices identified differences between the correlation matrix of student data and practitioner data, with item correlations notably lower and more diffuse in the practitioner group. As such, we decided to analyse the groups separately.

Items were removed due to low loading on individual items (i.e. < .30) and where ‘cross-loading’ across two or more items in both student and practitioner analysis had a difference of approximately .20 or less [12–14]. This process resulted in identifying a 23-item student inventory (QFI-S) with three components and a 23-item practitioner inventory (QFI-P) with four components for subsequent EFA. Table 1 presents the initial items and excluded items for the practitioner inventory and Table 2 presents the initial items and excluded items for the student inventory.

For the final stage, Principal Axis Factoring was selected as the method for EFA as assumptions of normality were violated [16]. As with the PCA, direct oblimin rotation strategy was used. Examination of the scree plot for the practitioner inventory suggested between 4 and 6 factors. Using Kaiser’s criterion (retain factors with eigenvalues > 1) four factors explaining 58.8% of the variance. This process was repeated for the student inventory. The scree plot showed inflexions that would support 3–5 factors. Retaining factors with eigenvalues > 1 extracted three factors explaining 67.5% of the variance. The pattern matrix for each inventory is provided in Table 3 (practitioner) and Table 4 (student) No additional items were removed from either inventory based on this analysis.

**Table 3:** Pattern matrix and communalities (*h*^2^) for practitioner inventory

Items		Pattern Matrix				*h*^2^
		F_1_	F_2_	F_3_	F_4_	
11.9	Feedback encouraged the student to think about what motivates them to learn	**0.731**	0.061	0.113	− 0.104	.591
2.1	The learning goals were agreed in advance with the student	**0.699**	− 0.095	− 0.053	− 0.024	.501
7.4	Feedback was expected	**0.686**	0.073	− 0.081	0.195	.444
11.7	Feedback encouraged the student to share their feelings about different experiences	**0.680**	0.140	0.051	−0.065	.573
5.4	Feedback occurred at an agreed time^a^	**0.667**	−0.100	− 0.034	− 0.068	.514
3.2	Feedback was informed by multiple sources	**0.488**	−0.027	−0.129	− 0.058	.388
8.2	Learning goals were modified based on feedback	**0.484**	−0.069	−0.012	− 0.246	.395
9.1	I felt the student was comfortable sharing their viewpoints^a^	**0.449**	0.034	−0.277		.400
10.4	The feedback I shared was non-threatening	−0.031	**0.768**	0.054	0.044	.531
10.3	The feedback I shared was non-judgemental^a^		**0.743**	−0.158	0.019	.632
10.1	The feedback I shared was respectful^a^	0.030	**0.715**	−0.050	−0.110	.635
5.3	Feedback was timely	0.158	0.054	**−0.606**	0.020	.492
7.1	The amount of feedback was manageable	0.022	0.112	**−0.591**	− 0.045	.445
5.1	There was enough time for feedback^a^	0.157	0.096	**−0.505**	−0.088	.465
7.2	Feedback was regular	0.207	−0.050	**−0.443**	− 0.288	.540
10.2	The feedback I shared was clear^a^	0.028	0.205	**−0.384**	(−0.324)	520
1.1	I encouraged the student to evaluate their practice	0.224	−0.016	0.030	**−0.612**	.539
8.4	Feedback helped the student to know how to improve their practice	−0.087	−0.015	− 0.296	**−0.581**	.488
6.6	I encouraged the student to ask questions to help them understand the feedback	0.154	0.247	0.218	**−0.568**	.522
6.2	Feedback relevant to the student’s situation^a^		0.181	−0.227	**− 0.561**	.609
2.2	Feedback was related to on workplace or university standards	0.025	0.021	−0.265	**−0.556**	.534
6.3	I encouraged the student to be involved in feedback conversations^a^	0.287	0.110	−0.046	**−0.457**	.537
10.6	I considered the emotional needs of the student^a^	0.261	0.154	0.013	**−0.341**	.365
Eigenvalues		8.94	1.85	1.45	1.29	
% variance explained		38.87%	8.04%	6.33%	5.59%	
Kaiser-Meyer-Olkin (KMO) measure of sampling adequacy		.902				
Bartlett’s test of sphericity		*p* < .001				
Cronbach’s alpha		.854	.798	.800	.855	
F_1_ – Collaborative preparation for feedback (*M =* 3.73, *SD =* 0.60)						
F_2_ – Imparting feedback (*M =* 4.65, *SD =* 0.47)						
F_3_ – Environmental context for feedback (*M =* 3.86, *SD =* 0.57)						
F_4_ – Learner-focused feedback (*M =* 4.29, *SD =* 0.57)						

Extraction Method: Principal Axis Factoring with Oblimin rotationLoadings ≥ .01 presented in pattern matrix

^a^ Items occurring in both student and practitioner inventory

Lower cross-loading items indicated in parentheses

**Table 4:** Pattern matrix and communalities (*h*^2^) for student inventory

		Pattern Matrix			*h*^**2**^
		F_1_	F_2_	F_3_	
3.1	Feedback was informed by observation of my practice	**0.688**	0.112		.589
6.2	Feedback was relevant to my situation^a^	**0.729**	0.017	0.108	.655
6.7	I understood what the feedback meant	**0.806**	− 0.109	0.138	.682
8.3	Feedback motivated me to change	**0.718**	−0.105	0.086	.502
8.4	Feedback helped me to know how to improve my practice	**0.691**	0.018	0.174	.670
9.1	I felt comfortable sharing my opinion/viewpoint^a^	**0.719**	0.174	−0.098	.612
9.3	Feedback was communicated in a way I understood	**0.793**	−0.085	0.109	.653
10.1	Feedback was respectful^a^	**0.849**	0.052	−0.122	.665
10.2	Feedback was clear^a^	**0.733**	0.123	−0.068	.603
10.3	Feedback was non-judgemental^a^	**0.661**	0.021	0.144	.592
10.5	Feedback focused on my practice	**0.609**	0.189	0.112	.671
2.3	Feedback related to specific standards	−0.080	**0.806**	0.124	.701
5.1	There was enough time for feedback^a^	0.216	**0.598**	0.010	.578
5.4	Feedback occurred at an agreed time^a^	−0.096	**0.766**	0.173	.675
6.3	I was encouraged to be involved in feedback conversations^a^	(0.303)	**0.490**	0.050	.570
6.5	I had the opportunity to clarify feedback	(0.444)	**0.496**	−0.026	.694
7.3	Feedback was planned	−0.079	**0.809**	0.121	.702
10.6	My emotional needs were considered^a^	0.294	**0.608**	−0.061	.620
11.1	Feedback was offered in more than one way	0.106	**0.660**	0.039	.573
1.2	I was encouraged to reflect on evaluation	0.046	0.080	**0.704**	.616
8.1	Learning goals were reviewed based on feedback	0.087	0.030	**0.631**	.497
11.4	Feedback focused on my knowledge	0.025	0.086	**0.717**	.620
11.6	My decision making process was considered	0.090	0.094	**0.690**	.656
Eigenvalues		12.43	1.86	1.23	
% variance explained		54.03%	8.07%	5.36%	
Kaiser-Meyer-Olkin (KMO) measure of sampling adequacy		.948			
Bartlett’s test of sphericity		*p* < .001			
Cronbach’s alpha		.946	.930	.857	
F_1_ – Individualised growth-oriented feedback (*M =* 4.39, *SD =* 0.62)					
F_2_ – Environmental context for feedback (*M =* 3.95, *SD =* 0.84)					
F_3_ – Goal-oriented feedback (*M =* 4.10, *SD =* 0.73)					

Extraction Method: Principal Axis Factoring with Oblimin rotationLoadings ≥ .01 presented in pattern matrix

^a^ Items occurring in both student and practitioner inventory

Lower cross-loading items indicated in parentheses

Four latent factors that emerged from the practitioner inventory were labelled: *Collaborative preparation for feedback* (eight items); *Imparting feedback* (three items); *Environmental context for feedback* (five items); and *Learner-focused feedback* (seven items). An explanation of each QFI-P factor is outlined in Table 5. In this inventory, item 10.2 (‘the feedback I shared was clear’) cross-loaded on factors three and four (refer to Table 3). Attempts to remove this item destabilised the pattern matrix. Therefore, it was retained in factor three as this had the higher loading.

**Table 5:** Description of factors in the Quality Feedback Inventory for students (QFI-S) and the Quality Feedback Inventory for practitioners (QFI-P)

Inventory	Factor	Explanation
QFI-S	Individualised growth-oriented feedback	The multiple components that guide the learner toward change (and growth) in their practice
	Environmental context for feedback	The immediate contextual factors to be considered for effective feedback encounters
	Goal-oriented feedback	The elements that assist a learner understand expected or desired goals
QFI-P	Collaborative preparation for feedback	The shared and invitational approach to encourage active participation within a feedback encounter
	Imparting feedback	The professional skills and manner that are used in feedback encounters
	Environmental context for feedback	The contextual considerations that support processes to establish and sustain feedback encounters
	Learner-focused feedback	The considerations to assist the learner comprehend the message of the feedback encounter

In contrast, three latent factors surfaced for the student inventory. These were labelled: *Individualised growth-oriented feedback* (11 items); *Environmental context for feedback* (eight items); *Goal-oriented feedback* (four items). A description of each of the QFI-S factors are presented in Table 5. Items 6.3 (‘I was encouraged to be involved in feedback conversations’) and 6.5 (‘I had the opportunity to ask questions’) in the student inventory cross-loaded on factors one and two and was retained in factor one due to the higher loadings (refer to Table 4). As with the practitioner inventory, item removal destabilised the pattern matrix.

Nine of the 23 items occurred in both the student and practitioner inventories (Table 3 and Table 4) and were distributed differently across the each of the factors demonstrating participants’ congruent and divergent perspectives of feedback. For example, items that reflected the concept of ‘Environmental context for feedback’ occurred in both the student and practitioner inventories although were represented by different items. All key attributes of effective feedback were expressed in the items of the practitioner and student inventories except for one attribute—‘Desired’ (i.e. feedback is welcomed and invited).

The Cronbach’s alpha coefficient (α) for the 23-item practitioner inventory was .926 and for the 23-item student inventory was .958—demonstrating good internal consistency. The Cronbach’s alpha coefficient for factors in each inventory are provided in Table 3 and Table 4.

## Discussion

As a mathematical method, factor analysis not only seeks to reduce the number of variables (in this example from more than 45 items to 23) but has the capacity to aid in data interpretation with each cluster of items representing a specific latent factor [17, 18]. However, beyond the ability of factor analysis to undertake structural analysis of the particular phenomenon (for example views of feedback) and instrument validation, is its value in abductive inference [17]. Early work by Shank identifies that “good abductive reasoning [inference] leads neither to the ridiculous, nor to the obvious. Instead, it leads to areas where we need further understanding” [19] (p.7). Therefore, we posit that ‘sense-making’ of resultant factors is the crucial next step in factor analysis and not solely reporting results of participant’s responses.

Our results provide preliminary empirical evidence of validity for the student and practitioner inventories. Through use of psychometric analysis, this study identified clustered items regarding how feedback is viewed and understood by students and practitioners in clinical placements in Australia. The results indicate that the factors within the QFI-S and QFI-P (Table 3 and Table 4) fit the data well, providing evidence of feedback constructs aligned with current conceptualisation of feedback [5, 6, 9]. Subsequently, these inventories support exploration of shared feedback encounters between learners and learning partners instead of merely a one-sided determination of satisfaction with feedback [1, 5]. Importantly, while the items were clustered differently across student and practitioner groups, items retained in both groups were representative of the empirically derived attributes of effective feedback. Thus, verifying the importance of each of the attributes identified in the literature [9] and resulting factors of the QFI-P and QFI-S derived through psychometric analysis.

Furthermore, these findings reflect that of a study by Adcroft [3] who found students and academics have different perceptions of feedback creating dissonance as each group offer divergent interpretations of feedback events. If we position ourselves with the assumption that we all have the same understanding of the term ‘feedback’ then this finding could be surprising. However, from a socio-constructive lens—where meaning is constructed based on our experiences of life and the world, and dialogue with others [20], coupled with each feedback encounter being unique for that situation and/or person—differences in item importance for each group is not unexpected. This difference in the importance of how each item is viewed by each group can be seen not only in the small number of over-lapping items, but through the clustering and the collective concepts represented in the identified factors.

When we return to the purpose of feedback—namely, to assist learners toward developing evaluative judgement (implicit within this rationale is that in developing evaluative judgement learners are better able to reach their required goals) it is arguably appropriate that learners view feedback through the lens of focusing on outcomes related to individual growth and goal attainment [3, 21]. In contrast, learning partners view feedback as organised around the concept of collaborative processes. As contemporary literature advocates for increasing student engagement to examine, reflect, and form an evaluation [22] then these parallel views may arguably coalesce well; vis-à-vis learning partners assisting processes for learners to attain their goals.

Substantive interpretation of the statistical factors determined three factors in the QFI-S and four factors in the QFI-P. Items included in the QFI-S reveal a strong outcome focus; for example ‘feedback helped me to know how to improve my practice (item 8.4) and ‘feedback focused on my knowledge’ (item 11.4). This focus towards outcomes is mirrored/comparable in student perspective pertaining to assessment (academic or WBA) [23, 24]. This furthermore supports the position that students view feedback from an outcome lens compared to practitioners. Divergence between students and practitioners is also observed in the factor *environmental context for feedback*. Considered collectively, QFI-S items grouped within this factor capture the immediate context in which feedback occurs. However, the grouping of items in the QFI-P for this factor establish the broader climate which buttresses the feedback encounter and message. Looking beyond individual items of each inventory sees the respective factors coalesce to form what could be called a harmonious dissonance. That is to say, despite the differing perspectives of the student and practitioner, when coupled together cohesive and functional understanding of feedback can result.

While a detailed discussion is outside the scope of this manuscript, differences seen in the correlation matrices of comparable items for each group raises some interesting points for consideration. Results indicated a greater number of items with very low item correlation coefficient values (≤ .2) observed in the practitioner group compared to the student group. The difference may be attributed to the potential for variations in familiarity and immersion in feedback and differences of feedback literacy—particularly in the practitioner group. We postulate that students are more accustomed with feedback terminology and place a higher value on seeking feedback to achieve their desired outcome from their program of study. This is in comparison with that of practitioners who support student learning in the workplace in conjunction with a priority to ensure quality patient outcomes and safe practice. This is thought-provoking given that two thirds (*n* = 122) of the practitioner group held an undergraduate bachelor’s degree in nursing (and exposed to ‘academic’ or ‘critical thinking’ language) and 77 practitioners had been practicing as a nurse for an average of 5 years (and therefore would have undergone very similar education to the student participants).

Access to logical approaches toward preferred feedback can help progress the adoption of feedback into practice. Valid sources can be the impetus to enact change where most needed (either for the learner or learning partner) and provide development opportunities for the learner or learning partner to enhance feedback ‘culture’ in their specific learning setting. It is important that merely clustering of items is not the sole consideration for inclusion in the development and validation of instruments, but the significance and meaning of these clustered items have within the context under exploration is also considered. This is evident in the exclusion of items that represent the attribute ‘desired’; for example, item 4.1 ‘I asked for feedback/‘I encouraged the student to ask for feedback’. While these items were excluded due to the mathematical methods of factor analysis, the items remain a central element of effective feedback [25] and warrant asking in any evaluation of feedback.

### Limitations

Despite the factors being underpinned by feedback concepts presented in the wider international literature and multiple disciplines, participants in our study were restricted—representing practicing nurses and student nurses from just one university and two health care facilities in Australia. This may contribute to a decreased ability to generalise the results beyond these settings. The items were explored in workplace-based settings where verbal feedback is the prevailing approach. It is recommended that future development of items includes engaging learners (e.g. students) in the consultation process to elicit more feedback behaviours important for learners in attaining their goals. Additionally, because completion of the lists of items was anonymous, there is no way of being able to use the data to help individuals improve feedback practices or recognise the performance of feedback encounters.

## Conclusions

Future research is needed to explore the differences observed between the student and practitioner groups and the possible impact these differences have on engagement with feedback and feedback literacy and dissonance between the learner and learning partner. The possible effects organisational culture has on the structure of feedback perceptions also warrants further research. Although development of the QFI started with a list of comparable statements, psychometric testing demonstrated minimal overlap of items between students and practitioners and resulted in two inventories—the QFI-S and the QFI-P. This divergence revealed a goal-oriented outcome focus for students and a process driven focus for practitioners. While this may appear to ‘fly in the face’ of dialogic feedback, congruent views are demonstrated through practitioners’ consideration of collaborative preparation and environmental context to undergird imparting learner-focused feedback that guides students towards their desired goals and outcomes for subsequent individual growth.

Simultaneous evaluation of both perspectives of feedback is not overtly evident in the literature and is needed to understand this issue further. When used in tandem, the QFI-P and QFI-S identify feedback encounters shared by the learner and learning partner. Information obtained from student and practitioner concurrent completion of both inventories has the potential to constructively inform feedback processes and thereby optimise the value of routine feedback. Additionally, this information may provide advice to adapt individual’s feedback practices to optimise feedback relationships, learning outcomes, and life-long learning.

## Supplementary Information

**Additional file 1:** **Supplemental material 1.** – Descriptive statistics of shared items by participant type

## Data Availability

Relevant data are included within the article. The raw data are not available for sharing due to confidentiality agreements approved by the Human Research Ethics Committee.

## Abbreviations

QFI: Quality feedback inventory
QFI-S: Quality feedback inventory-student
QFI-P: Quality feedback inventory-practitioner
PCA: Principal components analysis
EFA: Exploratory factor analysis

## References

[1] Bing-You R, Ramesh S, Hayes V, Varaklis K, Ward D, Blanco M (2018). Trainees' Perceptions of Feedback: Validity Evidence for Two FEEDME (Feedback in Medical Education) Instruments. Teach Learn Med.

[2] Paul A, Gilbert K, Remedios L, Boud DJ, Molloy EK (2013). Socio-cultural Considerations in Feedback. Feedback in Higher and Professional Education.

[3] Adcroft A (2011). The Mythology of Feedback. High Educ Res Dev.

[4] Bowen L, Marshall M, Murdoch-Eaton D (2017). Medical Student Perceptions of Feedback and Feedback Behaviors Within the Context of the "Educational Alliance". Acad Med.

[5] Halman S, Dudek N, Wood T, Pugh D, Touchie C, McAleer S (2016). Direct Observation of Clinical Skills Feedback Scale: Development and Validity Evidence. Teach Learn Med.

[6] Boud D, Molloy E (2013). Rethinking Models of Feedback for Learning: The Challenge of Design. Assess Eval High Educ.

[7] Yang M, Carless D (2013). The Feedback Triangle and the Enhancement of Dialogic Feedback Processes. Teach High Educ.

[8] Streiner DL, Norman GR, Cairney J (2015). Health Measurement Scales: A Practical Guide to Their Development and Use.

[9] Ossenberg C, Henderson A, Mitchell M (2019). What Attributes Guide Best Practice for Effective Feedback? A Scoping Review. Adv Health Sci Educ.

[10] DeVellis RF (2017). Scale Development: Theory and Applications.

[11] Dimitrov DM (2011). Statistical Methods for Validation of Assessment Scale Data in Counseling and Related Fields.

[12] Tabachnick BG, Fidell LS (2014). Using Multivariate Statistics.

[13] Field A (2018). Discovering Statistics Using IBM SPSS Statistics.

[14] Polit DF (2010). Statistics and Data Analysis for Nursing Research.

[15] Fabrigar LR, Wegener DT (2011). Exploratory Factor Analysis.

[16] Costello AB, Osborne JW (2005). Best Practices in Exploratory Factor Analysis: Four Recommendations for Getting the Most From Your Analysis. Pract Assess Res Eval.

[17] Haig BD (2018). The Philosophy of Quantitative Methods. The Oxford Handbook of Quantitative Methods.

[18] Pett MA, Lackey NR, Sullivan JJ (2003). Making Sense of Factor Analysis: The Use of Factor Analysis for Instrument Development in Health Care Research.

[19] Shank G (1987). Abductive Strategies in Educational Research. Am J Semiotics.

[20] Beck C, Kosnik C (2002). Components of a Good Practicum Placement: Student Teacher Perceptions. Teach Educ Q.

[21] Dawson P, Henderson M, Mahoney P, Phillips M, Ryan T, Boud D, Molloy E (2019). What Makes for Effective Feedback: Staff and Student Perspectives. Assess Eval High Educ.

[22] Nicol DJ, Macfarlane-Dick D (2006). Formative Assessment and Self-regulated Learning: A Model and Seven Principles of Good Feedback Practice. Stud High Educ.

[23] Massie J, Ali JM (2016). Workplace-based Assessment: A Review of User Perceptions and Strategies to Address the Identified Shortcomings. Adv Health Sci Educ.

[24] Nesbitt A, Baird F, Canning B, Griffin A, Sturrock A (2013). Student Perception of Workplace-based Assessment. Clin Teach.

[25] Hattie J, Timperley H (2007). The Power of Feedback. Rev Educ Res.

