# Supplementary material for: The use of factor analysis and abductive inference to explore students’ and practitioners’ perspectives of feedback: divergent or congruent understanding?
Source: BMC Med Educ. 2020 Nov 25;20:466. doi: 10.1186/s12909-020-02378-w (PMC7687844; doi:10.1186/s12909-020-02378-w)
Supplement: Supplementary file 1 — Additional file 1: Supplemental material 1. – Descriptive statistics of shared items by participant type [file 12909_2020_2378_MOESM1_ESM.docx]

Supplemental material 1 – Descriptive statistics of shared items by participant type

| **Item** | | **Participant type** | **Mean** | **Median** | **Standard deviation** |
| --- | --- | --- | --- | --- | --- |
| 5.1 | There was enough time for feedback | Student | 3.97 | 4 | 1.106 |
|  |  | Practitioner | 3.42 | 3 | 0.861 |
| 5.4 | Feedback occurred at an agreed time | Student | 3.82 | 4 | 1.132 |
|  |  | Practitioner | 3.39 | 3 | 1.007 |
| 6.2 | Feedback was relevant to my situation | Student | 4.44 | 5 | 0.691 |
|  |  | Practitioner | 4.35 | 4 | 0.627 |
| 6.3 | I was encouraged to be involved in feedback conversations | Student | 4.21 | 4 | 0.925 |
|  |  | Practitioner | 4.35 | 4 | 0.755 |
| 9.1 | I felt comfortable sharing my opinion | Student | 4.28 | 4 | 0.871 |
|  |  | Practitioner | 3.88 | 4 | 0.791 |
| 10.1 | Feedback was respectful | Student | 4.52 | 5 | 0.698 |
|  |  | Practitioner | 4.68 | 5 | 0.546 |
| 10.2 | Feedback was clear | Student | 4.35 | 5 | 0.812 |
|  |  | Practitioner | 4.22 | 4 | 0.669 |
| 10.3 | Feedback was non-judgemental | Student | 4.36 | 5 | 0.877 |
|  |  | Practitioner | 4.55 | 5 | 0.599 |
| 10.6 | My emotional needs were considered | Student | 4.03 | 4 | 0.978 |
|  |  | Practitioner | 4.36 | 4 | 0.726 |
